# Proteomics-Based Identification of Candidate Exosomal Glycoprotein Biomarkers and Their Value for Diagnosing Colorectal Cancer

**DOI:** 10.3389/fonc.2021.725211

**Published:** 2021-10-19

**Authors:** Zujun Sun, Shurong Ji, Junlu Wu, Jiale Tian, Wenqiang Quan, Anquan Shang, Ping Ji, Weidong Xiao, Ding Liu, Xuan Wang, Dong Li

**Affiliations:** ^1^Department of Clinical Medicine, Shanghai Tongji Hospital, School of Medicine, Tongji University, Shanghai, China; ^2^Department of General Surgery, Shanghai Tongji Hospital, School of Medicine, Tongji University, Shanghai, China; ^3^Herman B Wells Center for Pediatric Research, Indiana University School of Medicine, Indianapolis, IN, United States; ^4^Department of Chemistry, Georgia State University, Atlanta, GA, United States; ^5^Department of Pharmacy, Shanghai Putuo People’s Hospital, School of Medicine, Tongji University, Shanghai, China

**Keywords:** colorectal cancer, exosome, receiver operating characteristic, fibrinogen beta chain, beta-2-glycoprotein

## Abstract

Early diagnosis and treatment of colorectal cancer (CRC) significantly improves the survival rate and quality of life. Here we screened for differences in glycoproteins associated with tumor-derived exosomes and validated their clinical value to serve as liquid biopsy biomarkers to diagnosed early CRC. Exosomes were extracted from paracancerous tissues, cancer tissues, and plasma. LC-MS/MS proteomic and glycoproteomics analyses were performed using an LTQ-Orbitrap Elite mass spectrometer. The differences in glycoproteins associated with exosomes of paracancerous tissues and cancer tissue were determined, and their levels in plasma exosomes were determined. Statistical analysis was performed to evaluate the diagnostic efficacy of exosome-associated glycoproteins for CRC. We found that the levels of fibrinogen beta chain (FGB) and beta-2-glycoprotein 1 (β2-GP1) in the exosome of CRC tissue were significantly higher compared with those of paracancerous tissues exosome. The areas under the receiver operating characteristic (ROC) curves of plasma exosomal FGB and β2-GP1 as biomarkers for CRC were 0.871 (95% CI = 0.786–0.914) and 0.834 (95% CI = 0.734–0.901), respectively, compared with those of the concentrations of carcinoembryonic antigen concentration [0.723 (95% CI = 0.679–0.853)] and carbohydrate antigen19-9 concentration [0.614 (95% CI = 0.543–0.715)]. Comprehensive proteomics analyses of plasma exosomal biomarkers in CRC identified biomarkers with significant diagnostic efficacy for early CRC, which can be measured using relatively non-invasive techniques.

## Introduction

Colorectal cancer (CRC) is the third most common malignant tumor and is a serious global threat to human health (1). Early diagnosis of CRC is clinically significant, because it significantly improves patients’ survival rate and their quality of life. For example, the 5-year survival rates are 90% for patients with early-stage CRC and 13.1% for those diagnosed with late-stage CRC. Unfortunately, the lack of symptoms and biomarkers for early-stage CRC mainly explains the inability to diagnose early CRC, which excludes the possibility to provide such patients with potentially life-saving treatment.

Available approaches for screening for CRC are mainly based on endoscopic analysis of the mucosae followed by biopsy and fecal occult blood test (FOBT). These techniques are inherently limited, particularly for early diagnostic. Furthermore, endoscopic exams are invasive, costly, and associated with discomfort and procedural risk. Although the FOBT is non-invasive and affordable, its insufficient sensitivity and specificity prevent its use as a stand-alone diagnostic test (2, 3). Serum biomarkers such as carcinoembryonic antigen (CEA) and carbohydrate antigen 19-9 (CA19-9) are considered among the best available prognostic markers for CRC (3, 4). However, their low sensitivity and specificity limit their use as biomarkers for early diagnosis, and their expression levels are only applied for post-resection monitoring of patients already diagnosed with cancer. Certain new serological biomarkers such as non-coding RNAs are being evaluated for their clinical diagnostic value. Therefore, non-invasive, rapid, simple, and effective biomarkers for early diagnosis and for monitoring of the prognosis of patients with CRC are urgently required.

Recently developed as biomarker, exosomes are attracting much attention. Exosomes, which are derived from blood cells, dendritic cells, tumor cells, and other sources under physiological and pathological conditions, comprise complex membrane packets containing molecules such as miRNAs, mRNAs, lncRNAs, proteins, and bioactive lipids (5, 6). Exosomes released from donor cells into the cancer microenvironment affect the functions of target cells (6, 7). Their variations in abundance and half-lives in all biological fluids contribute to the potential of exosomes to serve as a source of biomarkers for early diagnosis, monitoring, and prognosis of patients with cancer. Evidence suggests that cancer-derived exosomes may contribute to tumorigenesis and metastasis as well as serving as biomarker (8, 9). Moreover, their presence in most body fluids makes exosomes potential candidates as clinical biomarkers for the early detection of different cancers, particularly because relatively non-invasive techniques can be used for this purpose (10–14). Most studies on exosomes as biomarker focused on associated nucleic acids, which can be amplified *in vitro* to enable high sensitive detection. Furthermore, the glycoprotein-associated exosomes may serve as biomarkers that can be readily measured using available detection technologies.

Protein glycosylation affects protein conformation, stability, spatial conformation, biological activity, transport, and localization required for diverse biological processes such as molecular recognition, cellular communication, and signal transduction (15–18). Glycoproteins, which are covalently linked through glycosidic bonds to oligosaccharides, are associated with the pathogenesis and progression of infectious diseases, tumors, cardiovascular disease, liver disease, kidney disease, diabetes, and certain genetic diseases (19–23). Moreover, glycoproteins on the cell surface are shed into the extracellular environment or enter the circulation and therefore can be used as biomarkers for abnormalities, which may be helpful for clinical diagnosis. The levels of certain glycoproteins in body fluids undergo disease-specific changes, which can be helpful for early diagnosis, guiding treatment, and prognosis. However, N-glycosylation proteins are present at low levels, which along with their structural complexity, makes them extremely difficult to detect. Therefore, it is a challenging but important task to detect and analyze the glycoproteins in plasma exosomes of patients with CRC.

Here we conducted proteomic and glycoproteomics analyses of three pairs of paracancerous tissues and their corresponding cancer-tissues exosomes. ELISA was used to detect selected glycoproteins associated with plasma exosome for the purpose of evaluating their potential to serve as biomarkers, with the ultimate goal to improve the early diagnosis of CRC.

## Materials and Methods

### Extraction of Tissue Exosomes

Banked frozen human tissue samples, including three pairs of paracancerous tissues and CRC tissues, were obtained from the Department of Gastrointestinal Surgery, Shanghai Tongji Hospital, Tongji University School of Medicine. Tissue samples were transported in ice and then added to 1640 medium precooled at 4°C. The tissues were cut into 1 mm^3^ pieces and kept on ice. Collagenase IV (350 μl, 1 mg/ml) and 2 μl of 0.2% (w/v) DNase I were added to the tissues, which were gently mixed, incubated in a constant temperature shaker (100 rpm/min) at 37°C for 60-90 min, and then stored 4°C. Processing time was ≤90 min. The tissue homogenates were then centrifuged at 3,000 × g at 4°C for 30 s, and the supernatant was centrifuged at 13,000 × g at 4°C for 10 min. The supernatant was passed through a 0.22 μm filter, mixed the PEG6000 (16% w/v) (1:1, v/v), gently mixed, incubated overnight at 4°C, and then centrifuged at 13,000 × g at 4°C for 30 min. The precipitate containing exosomes was centrifuged at 13,000 × g at 4°C for 5 min and suspended in.

### Extraction of Plasma Exosomes

Subjects granted their written informed consent for donating plasma (EDTA-K2) samples and pathological information. Plasma exosomes were prepared from blood samples of 30 patients with CRC enrolled between January 2016 and July 2016, as well as from 20 healthy individuals matched for sex and age (test and validation sets, respectively). Patients’ detailed clinical data are summarized in **Table 1**. Preoperative blood samples were collected into tubes containing an anticoagulant and centrifuged at 3,000 × g for 15 min at 4°C. The supernatant (250 μl) was added to a new tube, to which Exo-Quick™ solution (EXOQ5A‐1; SBI System Biosciences, USA) (63 μl) was added. The mixture was mixed, kept at room temperature for 30 min, and then centrifuged at 1,500 × g for 30 min. The supernatant was discarded, and the pellets were resuspended at 1,500 × g for 5 min. The pellets containing total exosomes were resuspended in 100 μl of phosphate-buffered saline (PBS).

**Table 1:** Characteristics of subjects (n.s., not significant).

Characteristics	Controls (n = 20)	CRC (n = 30)	p
Gender, n (%)	12 (60)	17 (56.7)	0.453
Smoking, n (%)	6 (30)	12 (40)	0.027
Drinking, n (%)	7 (35)	10 (33.3)	0.068
FGB (ng/L)	14.61 ± 3.12	24.34 ± 3.65	<0.01
β2-GP1 (ng/L)	23.46 ± 4.21	35.93 ± 5.61	<0.01
CEA (ng/ml)	3.40 ± 1.88	15.10 ± 5.80	0.021
CA19-9 (U/ml)	9.71 ± 3.52	18.96 ± 4.51	0.027

### Transmission Electron Microscopy

Isolated exosomes were resuspended in PBS, and 20 μl of the suspension was placed on a carbon-coated copper grid, which was incubated for10 min at room temperature. Next, the grid was washed using sterile distilled water, and 2% uranyl-oxalate solution was placed on the grids for 1 min and dried in air. The samples were observed using an electron microscope (JEOL-JEM1400, Tokyo, Japan).

### Nanoparticle Tracking Analysis

To measure the size and quantities of isolated particles, the suspension (1×10^7^/ml and 1×10^9^/ml) were examined using ZetaView PMX 110 (Particle Metrix, Meerbusch, Germany) equipped with a 405 nm laser. Videos were recorded (60 s, frame rate of 30 s), and particle movement was analyzed using NTA software (ZetaView 8.02.28).

### Western Blot Analysis

Exosome suspension was diluted with 5× sodium dodecyl sulfonate (SDS) buffer and was boiled for 10 min. Western blot analysis employed 10% SDS-polyacrylamide gel electrophoresis, 50 μg protein/lane. CD63 and TSG101 served as positive controls, and Calnexin served as a negative control. The rabbit polyclonal antibody CD63 (ab68418, 1:1000), TSG101 (ab30871, 1:1000), and Calnexin (ab22595, 1:1000) were purchased from Abcam (Cambridge, UK). After, samples were incubated with primary antibodies (overnight at 4°C), followed by the addition of an IgG goat anti-rabbit secondary antibody (1:2,000, A21020, Abbkine, Scientific Co., Ltd., Wuhan, China) for 1 h at 37°C. Immunocomplexes were detected using an enhanced chemiluminescence reagent (1856190; Thermo Scientific, USA).

### Proteomics and Glycoproteomics Analysis of Exosomes

Protein extraction: Exosomes were suspended in water, and proteins were precipitated using a solution containing chloroform: methanol: water (1:3:4, v/v). The middle layer containing a white precipitate of protein was washed twice with methanol.

Protein Digestion: Proteins were digested using FASP method and dissolved in 4% SDS, 50 mM DTT in 50 mM Tris-HCl (pH8.0). The solution was subsequently heated at 95°C water bath for 10 min, diluted with 8 M urea in 100 mM Tris-HCl, pH8.5 (UA solution) (final SDS concentration <0.5%), transferred to an Amicon 30-kD aultracentrifugal filter unit (MRCF0R030, Merck), and centrifuged at 14,000 × g for 30 min. Alkylation was performed by adding 50 μl of UA solution with 50 mM iodoacetamide to the filter unit, followed by incubation in the dark for 30 min at room temperature. After centrifugation at 14,000 × g for 10 min, 100 μl of UA solution was added to the filter unit, which was centrifuged four times. The filter unit was then washed three times with 100 μl of 50 mM NH_4_HCO_3_. Next, proteins were digested by adding 100 μl of 50 mM NH_4_HCO_3_ containing sequencing-grade trypsin (enzyme to protein ratio = 1:50) to the filter unit and incubating at 37°C for 14 h. Peptides were eluted using 100 μl of 50 mM NH_4_HCO_3_ and were collected by centrifugation at 14,000 × g for 10 min. This step was repeated five times. The peptides were further purified using a prepacked C18 ZipTip micro-column.

Glycopeptide enrichment: The enrichment of glycopeptides was performed using an iSPE HILIC cartridge. Briefly, the HILIC cartridge was prewashed with 300 μl of 0.1% TFA and equilibrated with 600 μl of 80% ACN containing 0.1% TFA. Peptide samples dissolved in 400 μl of 80% ACN containing 0.1% TFA were loaded onto the cartridge. The flow-through was reloaded onto the column twice, and the column was then washed with 1.2 ml of 80% ACN containing 1% TFA. The glycopeptides were sequentially eluted with 750 μl of 0.1% TFA, 60 μl of H_2_O, 60 μl of 25 mM NH_4_HCO_3_, and 60 μl of 50% ACN. The fractions were combined followed by lyophilization and stored at –20°C. Glycopeptide was then dissolved by 50 mM NH_4_HCO_3_ in ^18^O water and digested using PGNase F at 37°C for 16 h.

Proteomic and glycoproteomics analyses were performed using an LTQ-Orbitrap Elite mass spectrometer (ThermoFisher) equipped with an EASY-Spray source and a nano-LC UltiMate 3000 high-performance liquid chromatography system (Thermo Fisher). Each sample was separated using reversed-phase (RP)-HPLC fractionation on an EASY-Spray PepMap C18 column (length, 50 cm; particle size, 2 μm; pore size, 100 Å; Thermo Fisher), using a 120 min gradient as follows: 2 to 50% solvent B, flow rate of 300 nl/min (mobile phase A, 1.95% acetonitrile, 97.95% H_2_O, 0.1% formic acid). A full-scan survey MS experiment (*m/z* range from 375 to 1,600; automatic gain control target, 1,000,000 ions; resolution at 400 *m/z*, 60,000; maximum ion accumulation time, 50 ms) was performed using an Orbitrap mass analyzer. The 10 most intense ions were selected and fragmented in the LTQ mass spectrometer (automatic gain control target value, 10,000) *via* collision-induced dissociation (CID) with 100 ms maximum ion accumulation. Raw data were analyzed using Proteome Discoverer 1.4 (Thermo Fisher) to query the human Uniprot/TrEMBL database (2016_02 Release, 20,198 reviewed entries). Modifications were as follows: static modification of *via* carbamidomethyl (Cys, + 57.0214 Da); dynamic modification of glycosylation (Asn, + 2.9882 Da), oxidation (Met, + 15.9949 Da), and acetylation (Lys, + 42.0106 Da). Trypsin was selected as the proteolytic, and up to two missed cleavages were allowed. The mass tolerance was set 20 ppm for the precursor ions and 0.5 Da for the fragment ions. The false discovery rate = 1% for peptide and protein identification.

### ELISA Quantification of Glycoproteins

Exosomes were precipitated in 100 µl of RIPA lysis solution on ice for 30 min, oscillated, and fully mixed. The samples were diluted three times with 1×PBS. The levels of plasma exosomal FGB and β2**-**GP1 were determined using sandwich immunoassay after generating a standard curve using serial dilution of FGB and β2-GP1 (JL47995/JL19205, Jianglai Biotechnology Co., LTD, Shanghai, China). Briefly, samples (100 µl) were added to the ELISA plate, incubated for 60 min at 37°C, washed three times, after which solution B was added for 30 min at 37°C, plates were washed five times, followed by the addition of 90 µl of substrate, incubated for 15 min at 37°C, and addition 50 µl of termination solution. Absorbance at 450 nm was immediately measured.

### Statistical Analysis

Statistical analysis was performed using SPSS 19.0 statistical software (SPSS, Inc., Chicago, IL, USA) and GraphPad Prism 5.0. Exosomal glycoproteins were evaluated to identify patients of CRC and healthy individuals. Clinicopathological diagnoses served as the gold standard to assess the diagnostic significance of exosomal glycoprotein levels according to the results of receiver operating characteristic (ROC) curves analysis. *P* < 0.05 indicates a significant difference.

## Results

### Identification of Exosomes Extracted From Tissue and Plasma

**Figure 1:**
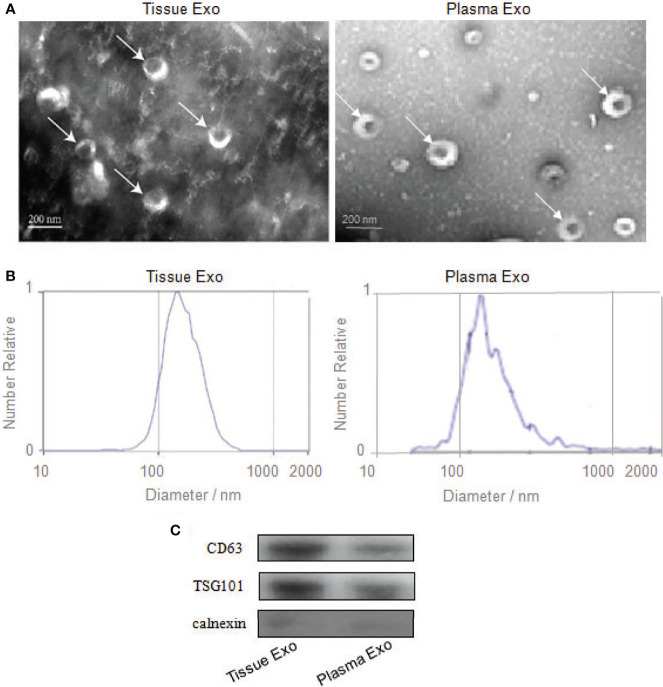
Identification of exosomes from tissue and plasma. **(A)** Transmission electron microscopy confirmed the presence of exosomes. Scale bar=200 nm. **(B)** Nanoparticle-tracking analysis determined the sizes of exosomes. **(C)** Western blotting analyzed the exosomes-enriched positive protein markers of CD63 and TSG101, and negative protein marker of Calnexin.

Subjects’ detailed information (30 patients with CRC and 20 healthy controls) is listed in **Table 1**. TEM analysis of plasma and tissue samples revealed the presence of round, cup-shaped, double-membrane-bound, vesicle-like structure (**Figure 1A**). NTA revealed that the diameters of spherical nanoparticles moving under Brownian motion ranged between 30 and 150 nm (**Figure 1B**). Western blot analysis of these samples detected the exosome markers CD63 and TSG101, but not the negative control, Calnexin (**Figure 1C**).

### Identification of Glycoproteins Specific for Colorectal Cancer

**Figure 2:**
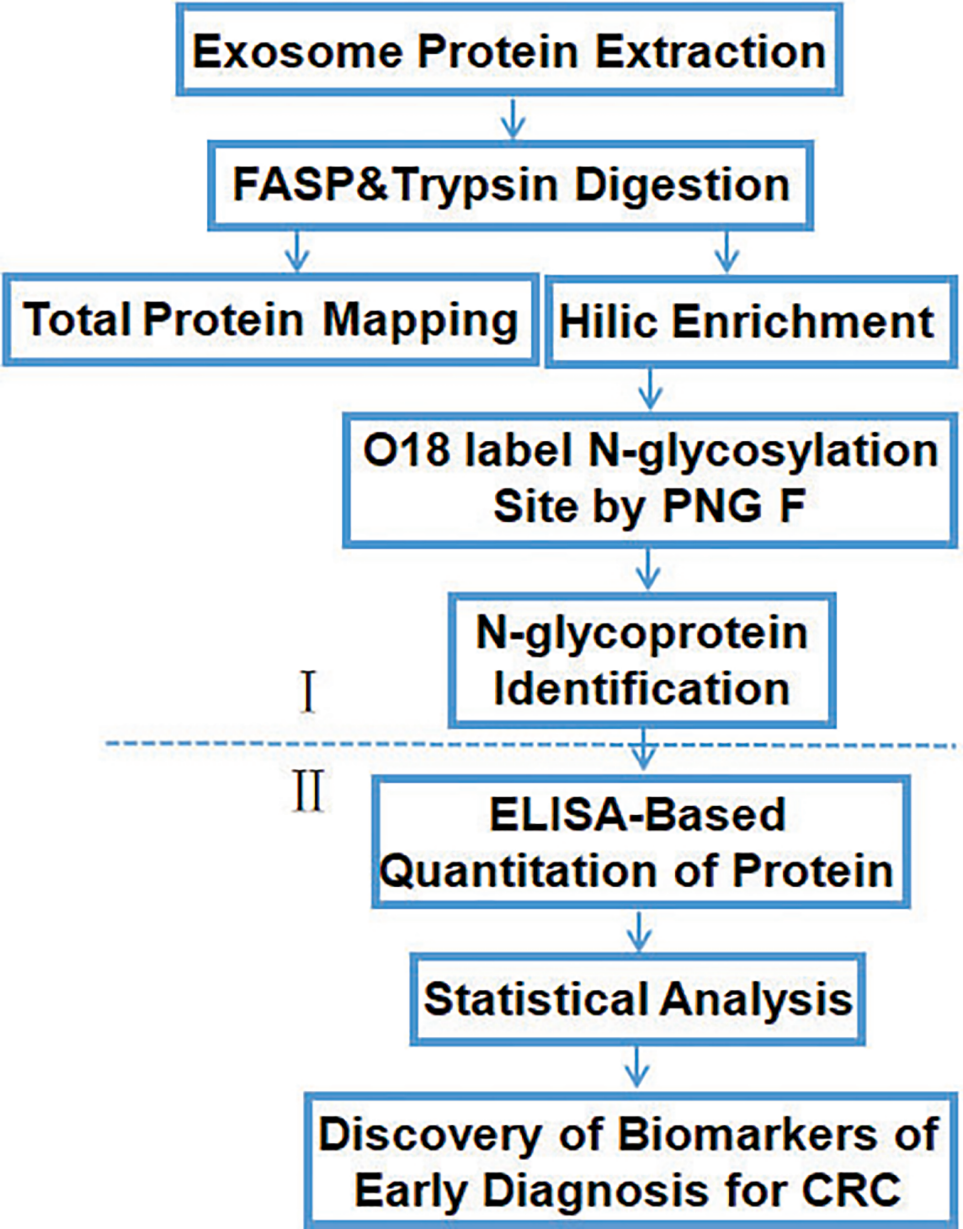
Workflow of the identification and quantification of exosomal glycoproteins in healthy controls and patients with CRC.

The workflow of the study (**Figure 2**) shows the screening (I) and verification (II) phase. In phase I, tissue exosomes were collected from patients with CRC and digested with trypsin for LC-MS/MS analysis. Database searches identified the corresponding glycoproteins. In phase II, ELISA was used to detect the levels of selected glycoproteins.

### Identification of Tissue Exosomal Total Proteins and Glycoproteins

**Figure 3:**
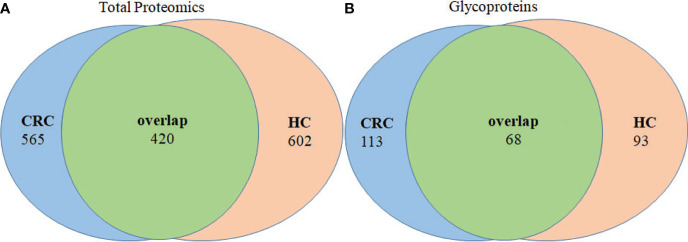
Venn diagram of proteins of paracancerous tissues and cancer-tissue exosome samples isolated from patients with CRC. **(A)** The distribution of total proteins and **(B)** unique glycoproteins.

Analysis of tissue exosomes pooled from three patients with CRC unambiguously identified 985 proteins in cancer tissues and 1,022 proteins in paracancerous tissue, among which 420 were identified in tissue exosomes of each source (**Figure 3A**). Furthermore, 565 and 602 proteins were unique to paracancerous tissue exosomes or cancer tissue exosomes, respectively. We unambiguously identified 181 glycoproteins in cancer tissue and 161 glycoproteins in paracancerous tissue, among which 113 and 93 glycoproteins, respectively, were unique (**Figure 3B**).

### Functional Classification of Glycoproteins of Tissue Exosomes

**Figure 4:**
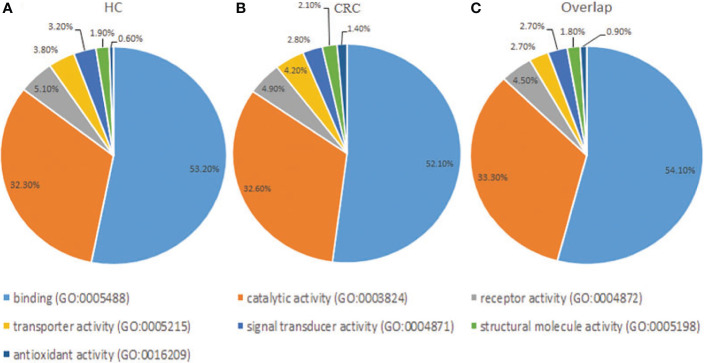
Gene Ontology analysis of the distribution of glycoproteins according to molecular function (http://exocarta.org/exosome_markers_new). **(A)** Paracancerous tissue exosomes (HC). **(B)** Cancer tissue exosome (CC). **(C)** Common cancer tissue and paracancerous tissue exosomes (Overlap). The frequencies of the glycoprotein functional categories are presented as percentages.

Gene Ontology analysis revealed that the frequencies of glycoprotein functions in paracancerous tissue exosomes were as follows: binding (53.20%), catalytic activity (32.30%), receptor activity (5.10%), transporter activity (3.80%), signal transducer activity (3.20%), structural molecule activity (1.90%), and antioxidant activity (0.60%) (**Figure 4A**). The functions of glycoproteins in cancer tissue exosomes were as follows: binding (52.10%), catalytic activity (32.60%), receptor activity (4.90%), transporter activity (4.20%), signal transducer activity (2.80%), structural molecule activity (2.10%), and antioxidant activity (1.40%) (**Figure 4B**). The functions of the majority of shared glycoproteins in tissue exosomes were as follows: binding (54.10%), catalytic activity (33.30%), receptor activity (4.50%), transporter activity (2.70%), signal transducer activity (2.70%), structural molecule activity (1.80%), and antioxidant activity (0.90%) (**Figure 4C**).

### Mass Spectrometry of Glycosylation Site in Glycoproteins in CRC Tissue Exosomes

**Figure 5:**
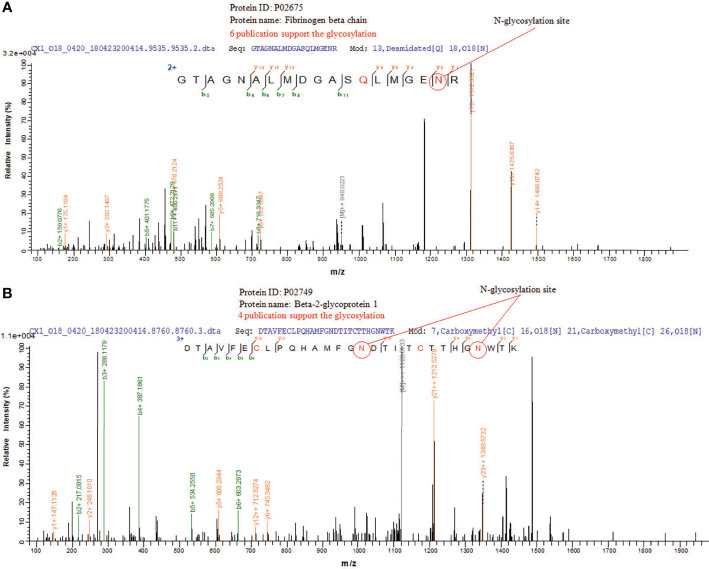
LC-MS/MS of N-glycosylations site of glycoproteins in CRC tissue exosomes. **(A)** Fibrinogen beta chain and **(B)** beta-2-glycoprotein 1.

N-glycosylation sites of tissue exosome samples were labeled using ^18^O during PNGase F digestion process. The asparagine (Asn) linked to glycan is converted to an aspartic acid residue paracancerous, and the oxygen atom in the hydroxyl moiety of the functional group of Asp is replaced with ^18^O. Thus, LC-MS/MS analysis detects a 2.9883 Da difference between glycosylated and unglycosylated Asp residues. Furthermore, through the CID fragmentation of the peptide, b and y ions are generated that confirm the peptide structure. **Figure 5A** shows the glycosylation of Asn394 of fibrinogen beta, consistent with six published data. **Figure 5B** shows the glycosylation of Asn183 and Asn193 of beta-2-glycoprotein 1, consistent with four published data.

### Elisa Analysis of the Glycoproteins as Biomarkers for Early Diagnosis of CRC

**Figure 6:**
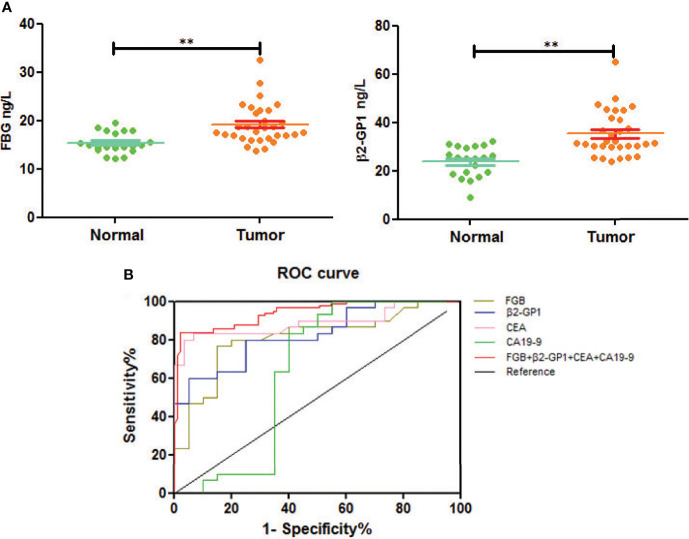
Verification of plasma exosomal glycoproteins as biomarkers for early diagnosis of CRC. **(A)** The levels of fibrinogen beta chain and beta-2-glycoprotein 1 in HC and CRC. **p < 0.01. **(B)** ROC curve of plasma exosomal fibrinogen beta chain and beta-2-glycoprotein 1.

We next used an ELISA to determine the glycoprotein levels of plasma exosomes of selected patients and controls. The levels of FGB and β2-GP1 were significantly higher in patients with CRC compared with those of healthy controls (p<0.01) (**Figure 6A**).

To understand if analyzing the levels of plasma exosomal glycoproteins served as diagnostic biomarkers, we evaluated the diagnostic efficacies of FGB and β2-GP1 in plasma exosomes and compared them with the values of CEA and CA19-9 (**Table 2**). The discriminatory power of each putative biomarker was further evaluated using (ROC) area-under-the-curve (AUC) analysis. The data show that the AUC value (0.871) of FGB directly isolated from plasma exosomes was higher compared with the values of serum CEA and CA19-9 (0.625 *vs.* 0.614). Furthermore, the AUC value of β2-GP1 (0.834) was higher compared with the values of CA19-9 and CEA. Moreover, combining the levels of the two plasma exosomal glycoproteins achieved a higher AUC compared with the values of CA19-9 and CEA (**Figure 6B**).

**Table 2:** ROC of plasma exosomal glycoproteins as biomarkers for early diagnosis of CRC.

marker	FBG	β2-GP1	FBG+β2-GP1	CEA	CA19-9
AUC (95% CI)	0.871	0.834	0.915	0.723	0.614
	(0.786–0.914)	(0.734–0.901)	0.845–0.987	(0.679–0.853)	(0.543–0.715)
Cutoff	18.6 ng/L	30.6 ng/L		4.7 ng/ml	27.0 U/ml
Sensitivity (%)	68.35	71.55	63.84	48.43	53.67
Specificity (%)	86.27	85.51	93.54	81.23	83.14
Positive likelihood ratio (%)	93.22	91.15	96.2	79.25	77.31
Negative likelihood ratio (%)	73.26	70.11	75.6	54.32	51.28

## Discussion

Upon diagnosis, the majority of patients with CRC present with advanced- to middle- and late-stage disease, mainly because of undetectable previous symptoms and the absence of specific biomarkers that detect early disease. Despite advances in clinical diagnosis and therapy, most patients experience very low survival rate. To address this serious problem, here we applied a novel targeted mass spectrometry proteomic approach to screen exosomal glycoproteins as potential biomarkers for early CRC. For example, no study, to our knowledge, reports the quantitation of differences in the abundances of exosomal glycoproteins between those of patients with CRC compared with controls, particularly using the specific combination of instruments.

In this study, we detected large amounts of lipids compared with those of proteins during extraction, and the CRC group had more glycoproteins than the control group (**Figure 3**). The associated targets of differentially expressed glycoproteins in cancer and paracancerous tissue exosomes were predicted, and their functional annotation was carried out using GO enrichment analysis. The results showed that they were involved in several potential biological pathways, including binding, catalytic activity, receptor activity, transporter activity, signal transducer activity, structural molecule activity, and antioxidant activity (**Figure 4**). The antioxidant activity was significantly different in paracancerous tissues and their corresponding cancer-tissue exosomes, indicating that the antioxidant activity of glycoproteins may be increased in patients with CRC. We identified glycosylated Asn394 of FGB and glycosylated Asn183 and Asn193 of β2-GP1 (**Figure 5**). Our protein identification and quantitation techniques are not high-throughput, and a relatively small sample set was used. Further verification of candidate glycoprotein markers was therefore required. For this purpose, we performed ELISA analysis, which confirmed that glycosylated forms of FGB and β2-GP1 were present at higher levels in the plasma exosome of patients with CRC compared with those of controls (**Figure 6A**).

The glycoprotein fibrinogen, which is synthesized and secreted mainly by hepatocytes, comprise three pairs of distinct polypeptide chains linked by disulfide bonds, termed α, β, and γ-chains (24). High fibrinogen levels serve as an important risk factor and clinical marker for thrombotic diseases. Furthermore, increased levels of plasma fibrinogen correlate with cancer metastasis, recurrence, and shorter survival (25). Plasma fibrinogen serves as an important tumor biomarker for cancers of the digestive tract, which is non-invasively measured, making it suitable for initial screening or combined with other biomarkers for cancer diagnosis or prevention (26).

The assessment of fibrinogen content and fibrinolysis product in plasma contributes to the diagnosis of cancer and the evaluation of therapy, tumor progression, tumor stage, and survival (27). FGB, which is cleaved to fibrin during the formation of blood clots, is present at higher levels in poor responders with rectal cancer, and a clinical validation study confirmed the predictive value of FGB (28). FGB levels significantly differ in the urinary tracts of patients with bladder cancer compared with those of controls and are elevated in bladder cancer tissue compared with those of morphologically normal tissue, indicating that FGB is a potential biomarker for bladder cancer (29).

Here we show that FGB-Asn394 is glycosylated, and its levels in patients with CRC significantly differed from those of healthy controls (**Figure 5**). Furthermore, plasma exosomal FGB achieved a higher value for diagnosis of early CRC compared with those of CEA and CA19-9 (**Figure 6B**).

The plasma glycoprotein β2-GP1 circulates in blood, primarily in free form, which contributes to triglyceride metabolism, blood coagulation, and homeostasis (30–32). Moreover, β2-GP1 inhibits apoptosis, LDL oxidation, and cholesterol accumulation in vascular cells, suggesting that β2-GP1 may regulate vascular functions (33, 34). β2-GP1 contributes to angiogenesis and is required to downregulate VEGF-induced cell growth and migration *in vitro* and *in vivo*, and inhibits the phosphorylation of VEGFR2, ERK1/2, and Akt (35). The circulating levels of β2-GP1 INHIBIT tumor growth and exert antiangiogenic effect on melanomas, bladder cancer, and prostate cancer, suggesting that β2-GP1 is a potential marker of the efficacies of angiogenesis-targeted therapy and diagnosis (36–39).

Here we detected the glycosylation of β2-GP1 residues Asn183 and Asn193 and found that β2-GP1 was present in higher levels in plasma exosomes of patients with CRC compared with those of controls. The levels of plasma exosomal β2-GP1 achieved higher efficacy for diagnosis of early CRC compared with those of CEA and CA19-9. Moreover, glycosylated FGB and β2-GP1 were identified to be in tissue exosomes and were present at higher levels in plasma exosomes of CRC compared with controls. Furthermore, FGB and β2-GP1 achieved higher sensitivity and specificity for the diagnosis of CRC compared with CEA and CA19-9. The cutoff values of the ROC curves (**Table 2**) reflect a trade-off between sensitivities and specificities. Additionally, we also analyzed the possibility of FGB and β2-GP1 as a panel to diagnose CRC. The results showed that the AUC value was markedly higher than FGB or β2-GP1 alone when discriminating CRC patients from controls (**Table 2**), suggesting the panel to be a better biomarker for CRC diagnosis.

There are limitations to the present study. First, a large amount of lipid during extraction may have affected the quality of the specimen and thus diminished the accuracy of the results, requiring further improvements in the method used to extract tissue exosomes. Second, the subject population comprising patients at multiple centers is required to support the application of standard liquid biopsy biomarkers for the diagnosis of early CRC. Third, the majority of the patients had advanced (T2–T4) disease. Future studies will therefore consider early-stage patients.

The combination of proteomic techniques and databases for screening and validation of plasma exosomal glycoproteins related to CRC shows that glycoproteins were enriched in tissue exosomes. The overexpression of FGB and β2-GP1 in patients with CRC compared with the control group achieved higher sensitivity and specificity for the diagnosis of CRC compared with the levels of CEA and CA19-9. FGB and β2-GP1 may therefore serve as biomarkers for diagnosing patients with early-stage CRC.

## Data Availability Statement

The original contributions presented in the study are included in the article/supplementary material. Further inquiries can be directed to the corresponding authors.

## Ethics Statement

The study was approved by the Ethics and Research Committee of Tongji Hospital and conducted according to the ethical guidelines of the 1975 Declaration of Helsinki.

## Author Contributions

ZS and SJ helped in the design of the research study and helped in acquiring data, analyzing data, and writing the manuscript. JT and JW helped in acquiring data, analyzing data, and writing the manuscript. WQ and AS helped in analyzing data and writing the manuscript. PJ and DiL helped in data acquisition and analysis. DoL, XW, and WX helped in the design and conception of the research study, data acquisition, and data analysis. All authors contributed to the article and approved the submitted version.

## Funding

This work was supported by the National Natural Science Foundation of China, Grant numbers 81974314 and 82072362; the Natural Science Foundation of Shanghai, Grant number 19ZR1448800; the Science and Technology Innovation Action Plan of Shanghai Science and Technology Commission, Grant numbers 19411964800 and 20YF1443700; Shanghai Public Health System Construction Three-Year Action Plan (2020-2022) Key Disciplines (GWV-10.1-XK04); the Clinical Research Project of Tongji Hospital of Tongji University [ITJ(ZD)1803, ITJ(ZD)1905].

## Conflict of Interest

The authors declare that the research was conducted in the absence of any commercial or financial relationships that could be construed as a potential conflict of interest.

## Publisher’s Note

All claims expressed in this article are solely those of the authors and do not necessarily represent those of their affiliated organizations, or those of the publisher, the editors and the reviewers. Any product that may be evaluated in this article, or claim that may be made by its manufacturer, is not guaranteed or endorsed by the publisher.
